# Climate-Driven Range Dynamics of the High-Altitude Frog *Nanorana parkeri* in Xizang, China: A Bias-Corrected, Multi-Algorithm Species Distribution Modelling Assessment

**DOI:** 10.3390/ani16142169

**Published:** 2026-07-13

**Authors:** Shi-Yang Weng, Cong Wei, Jian-Chuan Li

**Affiliations:** 1 Institute of Plateau Biology of Xizang Autonomous Region, Lhasa 850008, China; sws_wisely@sti.xizang.gov.cn (S.-Y.W.); sws_weicong@sti.xizang.gov.cn (C.W.); 2 Xizang Museum of Natural Science, Lhasa 850011, China

**Keywords:** species distribution model, sampling bias, CMIP6, amphibian conservation, Xizang, *Nanorana parkeri*

## Abstract

The climate sensitivity of high-altitude amphibians on the Qinghai–Tibet Plateau remains poorly quantified. We assessed habitat suitability for the plateau-endemic frog *Nanorana parkeri* across Xizang, China. Using 52 thinned occurrence records and nine predictors (spanning climate, terrain, and hydrological accessibility), we built a bias-corrected random forest (RF) model with VIF-screened variables, constrained complexity, and out-of-fold (OOF) evaluation, including spatial-block cross-validation. Gradient boosting (GBM) and a regularized GLM were fitted on identical data, and the RF was projected onto a two-GCM CMIP6 ensemble under four Shared Socioeconomic Pathways through 2100. The RF achieved OOF and spatial-block AUCs of 0.750 and 0.672 (GLM 0.734/0.691; GBM 0.692/0.584); all three converged on a current suitable area of 8.13 × 10^5^ km^2^. Under future climate, net habitat expansion reached 117–126% of current area by 2081–2100. Range turnover was asymmetric: ~96% remained stable, 3–5% was lost, and 17–29% was gained upslope. Inter-GCM uncertainty was low (SD 0.016–0.022), but MESS extrapolation was substantial (55–67% of Xizang). Net upslope expansion represents a robust climatic opportunity, but realised gains depend on persistent breeding waters. Conservation actions should prioritise protecting stable core breeding waters in Southern and Southeastern Xizang and monitoring upslope colonisation fronts.

## Simple Summary

The Qinghai–Tibet Plateau is warming faster than almost anywhere else on Earth, and we still know little about how its cold-adapted frogs will cope. This study asks where suitable habitat for a high-altitude frog, *Nanorana parkeri*, occurs across Xizang today, and how that habitat may shift as the climate warms through the end of the century. Using field and museum records, together with climate, terrain, and water-distance information, we built a carefully validated computer model that predicts where the frog can live, and we checked the result with three independent modelling methods so the conclusion does not depend on any single technique. We then projected the model onto future climate scenarios. Rather than shrinking, suitable habitat is predicted to expand modestly as warming opens up higher, formerly too-cold ground, while most current habitat remains intact. However, this frog can only breed where there is standing water, and the plateau’s lakes, streams, and glacier-fed wetlands are themselves changing. Our findings suggest that protecting existing breeding waters and watching whether the frog can actually move uphill are the most useful conservation actions, and that water availability, not temperature alone, may decide this species’ future.

## 1. Introduction

Amphibians are the most threatened vertebrate class globally, and climate change is increasingly implicated alongside habitat loss and disease as a driver of their decline [1,2]. Mountain and plateau endemics are of particular concern because warming is amplified at high elevations and because cold-adapted species occupying the upper limits of terrestrial habitat have limited capacity to track suitable conditions further upslope [3,4]. The Qinghai–Tibet Plateau, the highest and largest plateau on Earth, has warmed at roughly twice the global mean rate over recent decades, making its endemic fauna a natural laboratory for assessing the climate sensitivity of high-altitude ectotherms [5,6]. The second Global Amphibian Assessment confirmed that climate change affects an increasing proportion of species and is now the primary driver of status deterioration for nearly 40% of species whose extinction risk has worsened since 2004 [7].

*Nanorana parkeri* (Stejneger, 1927) (Anura: Dicroglossidae) is a small frog endemic to the Qinghai–Tibet Plateau, occurring largely above 2800 m and reaching some of the highest elevations recorded for any anuran. The species is closely tied to permanent and semi-permanent water bodies—lake margins, slow river reaches, marshes, and spring-fed pools—for breeding and overwintering. Its genome has been sequenced and it has become a model for studying high-altitude adaptation in amphibians [8], yet spatially explicit assessments of how its suitable habitat is distributed across the plateau and how that distribution may shift under future climate remain scarce. The genus Nanorana is a radiation of hypoxia-tolerant, high-elevation specialists endemic to the Himalayan–Tibetan orogen, whose diversification was driven by the uplift of the Qinghai–Tibet Plateau [9].

Species distribution models (SDMs) are the standard tool for such assessments, but they are vulnerable to several well-documented pitfalls that are especially acute for range-restricted, sparsely sampled species [10]. First, small occurrence samples combined with flexible machine learning algorithms invite overfitting, inflating apparent performance [11,12]. Second, opportunistic occurrence data—including the bulk of records aggregated in the Global Biodiversity Information Facility (GBIF)—are spatially biassed toward roads, settlements and research stations, so that a naive random background confounds the species’ niche with the geography of sampling effort [13]. Third, performance metrics computed on the training data, or thresholds chosen on fitted rather than cross-validated predictions, are optimistically biassed; spatially structured data further inflate random cross-validation unless folds are spatially blocked [14]. Fourth, projecting models into future climates frequently requires extrapolation beyond the environmental conditions represented in the training data, which standard accuracy metrics do not reveal [15,16]. Studies that ignore these issues can report impressively high AUC values while producing range forecasts that are essentially artefacts of sampling design. A further concern is algorithm dependence, as different SDM learners can yield divergent projections from identical data, so single-algorithm forecasts are increasingly regarded as insufficient and ensemble or multi-algorithm cross-checks are recommended [17,18]. Inconsistent reporting of modelling decisions has long hindered reproducibility, and a standard protocol for documenting SDM choices has recently been proposed [19].

Here we develop a deliberately conservative, bias-corrected habitat suitability model for *N. parkeri* across Xizang, China (hereafter Xizang)—the administrative jurisdiction responsible for the species’ conservation and monitoring—and use it to project climate-driven range dynamics through 2100. Our objectives are: (1) to identify the environmental correlates of habitat suitability for *N. parkeri* using a predictor set screened for collinearity; (2) to estimate current suitable habitat using a model corrected for sampling bias, overfitting, and evaluation optimism, and verify that the result is robust across three contrasting algorithms; (3) to project suitability onto a multi-GCM CMIP6 ensemble under four emission scenarios and quantify inter-model uncertainty and the degree of climatic extrapolation; and (4) to characterize the spatial pattern of range stability, loss, and gain. We explicitly favour honest, lower performance metrics over inflated ones, on the premise that defensible forecasts of the *direction* of change are more useful for conservation planning than precise but unreliable area estimates. This approach aligns with recommended standards for distribution modelling in biodiversity assessments [10].

## 2. Materials and Methods

### 2.1. Study Area

The study area is the Xizang Autonomous Region in Southwestern China, spanning 78.35–99.15° E and 26.81–36.52° N. Analyses were conducted on a regular geographic grid at 30 arc-second resolution (0.00833°, ~1 km) comprising 1165 rows × 2496 columns. After masking to the administrative boundary and to cells with complete environmental coverage, 1,643,341 grid cells (total area ~1.21 × 10^6^ km^2^) were retained for modelling. All area statistics were computed with a per-row cosine-latitude correction to account for meridian convergence.

**Figure 1:**
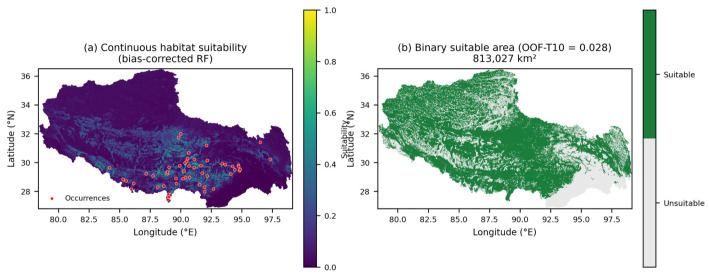
Current habitat suitability for *N*. *parkeri* across Xizang. (**a**) Bias-corrected random forest suitability surface, with the 52 modelling occurrence points overlaid. (**b**) Binary suitable area derived from the OOF-T10 threshold. This map is confined to the Xizang study area, which contains the species’ core high-elevation range and aligns with the management jurisdiction. *N*. *parkeri* also occurs above 2800 m in north-central Nepal and is expected in Bhutan and Sikkim (India), but confirmed georeferenced records from these regions are not available in GBIF or the primary literature (see Section 4.4).

This study was scoped to Xizang as a regional habitat-suitability assessment, because Xizang is the administrative unit within which conservation planning, protected-area management, and field monitoring of *N. parkeri* are actually carried out, and it contains the great majority of the species’ high-elevation core range. Aligning the modelling domain with the management jurisdiction means the suitability surfaces, threshold-based suitable areas, and projected range dynamics reported here map directly onto the spatial units used by regional decision-makers rather than onto a biogeographic range that crosses several jurisdictions. The original field records were also collected within Xizang. Two points of interpretation follow from this regional scope. First, all area statistics (e.g., current and projected suitable area) are properties of the Xizang assessment window and are not intended as estimates for the species’ entire plateau-wide range, part of which lies outside the region. Second, because the background and the MESS reference environment are drawn from within Xizang, suitability and climatic extrapolation are quantified relative to environmental conditions inside the region. We strengthen the regional inference by drawing the target-group background from plateau-wide amphibian sampling efforts (Section 2.4), and we frame the central conclusions in terms of the direction and spatial pattern of change within Xizang—the quantities most relevant to regional management—rather than absolute range-wide totals. An overview of the modelling framework, study-area map, and present-day suitable habitat surface produced by the final bias-corrected random forest is given in Figure 1. Figure 1 presents the bias-corrected RF suitability surface alongside the binary suitable/unsuitable classification (derived from the OOF-T10 threshold), with the 52 modelling occurrence points overlaid. The continuous suitability surface illustrates how the model distils the nine retained environmental predictors into a spatially explicit prediction of habitat quality, confirming the species’ known affinity for the wetter, warmer river valleys and lake basins of Southern and Southeastern Xizang. The binary map operationalises this continuous surface into a discrete suitable area, which serves as the baseline for all subsequent area statistics and future projections.

### 2.2. Occurrence Data

Occurrence records of *N. parkeri* were compiled from GBIF (taxon key 2430314, family Dicroglossidae; records spanning 1980–2024, downloaded in June 2026 as presence-only points with geographic coordinates; https://www.gbif.org/occurrence/search?taxon_key=2430314, accessed on 24 June 2026) and supplemented with 11 georeferenced field records collected during herpetological surveys by the authors (2018–2023); after spatial thinning at 10 km, 8 of these 11 were retained in the modelling dataset. GBIF records were cross-checked against the field dataset and duplicates (same locality within 1 km) were removed. Records were retained only if georeferenced, free of recorded geospatial issues, and fell within the study area. To reduce spatial autocorrelation and the influence of sampling clusters, records were spatially thinned at a 10 km separation distance. The final modelling dataset comprised 52 occurrence localities (44 from GBIF, 8 original field records) spanning 84.17–97.31° E and 27.43–32.00° N. We acknowledge that this is a small sample; the methodological choices below were designed specifically to remain defensible at this sample size. The original (pre-thinning) occurrence data showed pronounced spatial clustering in three major concentration areas—the Yarlung Zangbo River valley, the Lhasa River basin, and lake-margin wetlands of the southern plateau—reflecting both the species’ ecological association with valley–lake systems and the concentration of field survey effort along roads and research stations accessible from Lhasa. These clusters are characteristic of the sampling artefact addressed by the target-group background correction in Section 2.4. Across the 52 modelling points, elevation extracted from the WorldClim DEM at each occurrence point ranged from 3029 to 5930 m (mean 4332 m). The elevational distribution was concentrated at high altitudes: 16 points (30.8%) between 3000 and 4000 m, 33 (63.5%) between 4000 and 5000 m, and 3 (5.8%) above 5000 m. The eight retained field records spanned 3790–4766 m (DEM-extracted) and the 44 GBIF-derived records covered 3029–5930 m, ensuring that the model was informed by localities spanning the species’ full documented elevational range [8]. A complete list of the 52 modelling occurrence records with geographic coordinates, elevations, and data sources is provided in Table S1.

### 2.3. Environmental Predictors

We assembled an initial pool of climatic, topographical, and hydrological predictors. Climate variables were drawn from the standard 19 WorldClim bioclimatic layers [20]; topography from a digital elevation model (elevation, slope); and hydrological accessibility as Euclidean distance to the nearest river and to the nearest lake (km), derived from the HydroSHEDS drainage network [21]. Distance-to-water predictors were included because *N. parkeri* is an obligate water-breeder, whose distribution is expected to be constrained by surface-water availability beyond what climate alone captures.

To control multicollinearity (which destabilizes variable-importance estimates and response curves), we applied iterative VIF screening: the predictor with the highest variance inflation factor was removed and VIFs recomputed until all remaining predictors had VIF < 10. Elevation was removed at the first step (VIF = 28.8), being strongly collinear with the temperature variables on the plateau. The final predictor set comprised nine variables: annual mean temperature (bio01), mean diurnal range (bio02), isothermality (bio03), annual precipitation (bio12), precipitation of the driest month (bio14), precipitation seasonality (bio15), slope, distance to river, and distance to lake.

### 2.4. Sampling-Bias-Corrected Background

Instead of drawing background points uniformly at random—which would treat the heavily biassed spatial distribution of occurrence records as if it represented the niche—we built a target-group background [13]. Sampling effort was approximated by a two-dimensional Gaussian kernel-density surface fitted to the locations of all plateau amphibian records (the target group). Background points (n = 10,000) were then drawn from valid grid cells with selection probability proportional to this effort surface, so that the background reproduces the spatial bias of the occurrence data. This makes the contrast between presences and background reflect environmental preference rather than the geography of sampling.

### 2.5. Model Fitting and Multi-Algorithm Comparison

We fitted a random forest classifier (presence vs. background) with deliberately constrained complexity to limit overfitting at small sample size: 800 trees, maximum depth 8, minimum leaf size 3, and balanced subsample class weighting. A fixed random seed (42) ensured full reproducibility.

To test whether our conclusions were robust to the choice of statistical learner, we additionally fitted two contrasting algorithms on identical training data, predictors, and cross-validation folds: (i) gradient-boosted regression trees (GBM; 400 trees, maximum depth 3, learning rate 0.03, subsample fraction 0.75), the boosting analogue of the widely used boosted regression tree approach; and (ii) a regularized logistic GLM (standardized predictors, L2 penalty, balanced class weights), a parametric linear baseline. The three algorithms span the methodological spectrum from a flexible bagging ensemble (RF) through a regularized boosting ensemble (GBM) to a constrained linear model (GLM). Class imbalance was handled internally for RF (balanced subsample) and GLM (balanced class weights); the gradient-boosting implementation does not provide a class-weight argument, so its absolute probability scale and AUC should be interpreted cautiously. For spatial area comparison, however, each algorithm was binarized using its own OOF 10th-percentile training-presence (OOF-T10) threshold, a relative occurrence-based threshold that reduces dependence on raw probability scale. We compared algorithms on identical out-of-fold and spatial-block folds (Section 2.6) and quantified the spatial agreement of their continuous predictions (Spearman’s ρ over all valid cells) and of their binary suitable areas (the Jaccard index at each model’s own OOF-T10 threshold). A three-algorithm ensemble (per-cell mean suitability) was computed for visual comparison only. Future projections (Section 2.7) were generated from the bias-corrected RF, which combined the highest honest discrimination with an intermediate, ecologically interpretable suitable area.

### 2.6. Evaluation

All performance metrics were computed from out-of-fold (OOF) predictions to eliminate training-set optimism. We report: (i) the area under the ROC curve (AUC) from 5-fold stratified cross-validation and from pooled OOF predictions; (ii) the maximum True Skill Statistic (maxTSS) and its associated threshold, computed on OOF predictions; and (iii) the continuous Boyce index, the most appropriate metric for presence–background data [22]. Because random cross-validation overestimates transferability when data are spatially structured, we additionally performed spatial-block cross-validation [14], assigning 1° geographic blocks to five folds so that training and validation data are spatially separated. Binary suitable/unsuitable maps were produced using the OOF-derived 10th-percentile training-presence threshold (OOF-T10) and the OOF-maxTSS threshold.

### 2.7. Future Climate Projections

We projected the bias-corrected RF onto future climate using two CMIP6 general circulation models (GCMs): BCC-CSM2-MR and MIROC6 (WorldClim CMIP6 30 arc-second bioclimatic layers). Projections covered four Shared Socioeconomic Pathways (SSP1-2.6, SSP2-4.5, SSP3-7.0, and SSP5-8.5) and four time slices (2021–2040, 2041–2060, 2061–2080, and 2081–2100), i.e., 16 scenario–period combinations per GCM. Only six bioclimatic predictors varied between scenarios; the topographical and hydrological predictors (slope, distance to river, distance to lake) were held at present-day values, as future plateau-wide hydrological reconfiguration is not reliably available at this resolution. This assumption is discussed as a limitation.

For each scenario–period combination, we built a GCM ensemble by taking the per-cell median suitability across the two GCMs; inter-model uncertainty was summarized as the per-cell standard deviation, and model agreement as the number of GCMs classifying a cell as suitable. Suitable areas were computed with the OOF-T10 threshold and the same cosine-corrected areas accounting as the present. We classified each cell as stable (suitable now and in the future), lost (suitable now, unsuitable in the future) or gained (unsuitable now, suitable in the future).

To assess the reliability of projections, we computed a multivariate environmental similarity surface (MESS) [15] for each GCM and scenario relative to the occurrence-point environmental envelope, and report the fraction of the study area with negative MESS (i.e., requiring extrapolation beyond training conditions).

### 2.8. Software and Reproducibility

**Figure 2:**
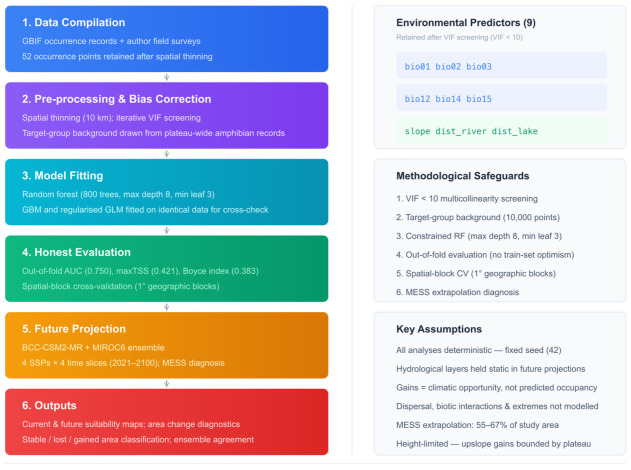
Schematic workflow of the species distribution modelling framework. The six-phase pipeline spans data compilation, preprocessing and bias correction, model fitting with multi-algorithm cross-check, honest evaluation, future projection, and output generation. Key methodological safeguards—VIF screening, target-group background, constrained RF complexity, out-of-fold evaluation, spatial-block cross-validation, and MESS extrapolation diagnosis—are annotated.

All analyses were implemented in Python (NumPy, scikit-learn, SciPy, rasterio). The complete workflow—data loading, model fitting, multi-algorithm comparison, evaluation, projection, and table generation—is deterministic given the fixed random seed and is available as a set of numbered, self-contained scripts (see Data Availability Statement). An overview of the modelling workflow is presented in Figure 2.

All maps use a latitude-corrected aspect ratio (1/cos(31.66°) ≈ 1.175), so that the Xizang outline is rendered without east–west distortion. Style is unified across figures: 600 dpi, consistent colour scales (viridis for suitability, RdBu for MESS, magma for uncertainty) and colourbar conventions.

## 3. Results

### 3.1. Environmental Correlates of Suitability

**Figure 3:**
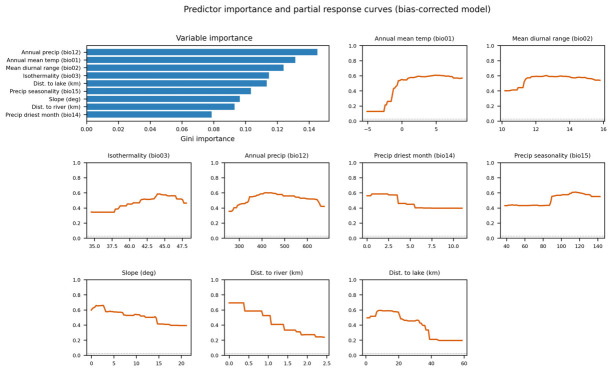
Variable importance (Gini index) and partial response curves for the nine retained predictors in the bias-corrected random forest model.

After VIF screening, the nine retained predictors contributed comparably to the random forest model, with no single variable dominating—consistent with a multifactorial niche. Ranked by Gini importance, the leading predictors were annual precipitation (bio12, 0.145), annual mean temperature (bio01, 0.131), mean diurnal range (bio02, 0.124) and isothermality (bio03, 0.115), followed by distance to lake (0.113) and precipitation seasonality (bio15, 0.103). The two hydrological-accessibility predictors together accounted for a Gini importance of 0.206, supporting proximity to surface water as a substantial, independent correlate of suitability beyond climate. The spatial distribution of the resulting suitable habitat, illustrated in Figure 1, and the variable-importance profile with partial response curves are shown in Figure 3 (Table 1).

### 3.2. Model Performance and Robustness Across Algorithms

Under honest out-of-fold evaluation, the bias-corrected random forest achieved a pooled OOF AUC of 0.750 and a 5-fold cross-validated AUC of 0.754 ± 0.041. The maximum TSS was 0.421 (at a suitability threshold of 0.090) and the continuous Boyce index was 0.383. Spatial-block cross-validation, the most demanding test of transferability, yielded an AUC of 0.672 ± 0.069. These values are modest by comparison with the inflated metrics typical of uncorrected small-sample SDMs; we regard them as the honest performance of a model that does not exploit sampling artefacts.

**Figure 4:**
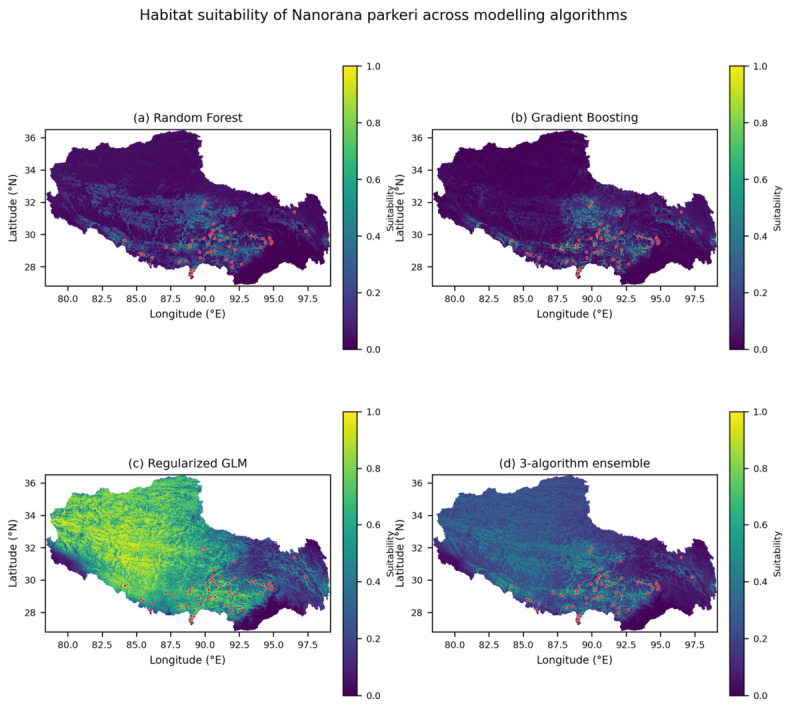
Habitat suitability of *N. parkeri* predicted by three algorithms fitted on identical data—(**a**) random forest, (**b**) gradient boosting, (**c**) regularized GLM—and (**d**) their three-algorithm ensemble.

The three algorithms, fitted on identical data and folds, told a consistent story. The random forest gave the highest pooled OOF AUC (0.750), the regularized GLM was close behind (0.734) with the best spatial-block AUC (0.691 ± 0.068) and the highest Boyce index (0.934), while the gradient-boosted trees were the weakest discriminator (OOF AUC 0.692; spatial-block AUC 0.584 ± 0.093) despite a high Boyce value (0.717). All three delineated broadly suitable landscapes at their own OOF-T10 thresholds: 67.5% of the region for RF (8.13 × 10^5^ km^2^), 67.6% for GBM (8.14 × 10^5^ km^2^), and 81.2% for the more permissive GLM (9.79 × 10^5^ km^2^). Spatially, RF and GBM predictions were strongly correlated (Spearman’s ρ = 0.708; binary Jaccard 0.648) and RF tracked the three-algorithm ensemble closely (ρ = 0.742); the linear GLM was the most divergent in continuous pattern (RF–GLM ρ = 0.466) yet still overlapped substantially in its binary suitable area (Jaccard 0.717). The convergence on a broad, water-linked suitable region across a bagging ensemble, a boosting ensemble and a linear model indicates that the core finding is a property of the data rather than of any single learner. We carried the random forest forward for projection because it combined the strongest honest discrimination with an intermediate, ecologically interpretable suitable area. The spatial distribution of habitat suitability predicted independently by each algorithm and by the three-algorithm ensemble is shown in Figure 4. Detailed performance metrics and pairwise concordance values are reported in Table 2 and Table 3, respectively.

### 3.3. Current Suitable Habitat

**Figure 5:**
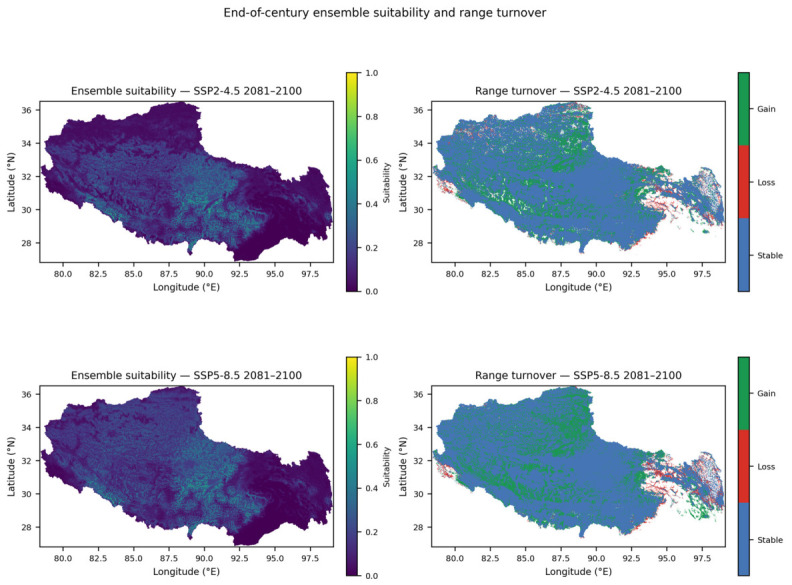
Ensemble median suitability and range turnover (stable/loss/gain) for end-of-century scenarios (SSP2-4.5 and SSP5-8.5, 2081–2100).

Applying the OOF-T10 threshold, the current suitable area for *N. parkeri* across Xizang was 8.13 × 10^5^ km^2^ (1,110,983 grid cells), or ~67% of the modelled region. The more restrictive OOF-maxTSS threshold delineated a core suitable area of 3.33 × 10^5^ km^2^. Suitable habitat was concentrated in the southern and southeastern plateau, associated with river valleys, lake basins, and the comparatively warmer, wetter conditions of these areas (Figure 5).

### 3.4. Projected Range Dynamics

Across all 16 scenario–period combinations, the two-GCM ensemble projected a net expansion of suitable habitat relative to the present. By the end of the century (2081–2100), the ensemble suitable area reached 117.3% of the current area under SSP1-2.6, 123.3% under SSP2-4.5, 126.3% under SSP3-7.0, and 126.0% under SSP5-8.5 (Table 4). Expansion generally increased with both time and emission intensity, though it began to plateau in the highest-emission scenarios late in the century.

Range turnover was strongly asymmetric (Table 5). Across scenarios and periods, ~95–97% of currently suitable habitat remained stable, only 3–5% was lost, and 17–29% of the current area was added as newly gained habitat. Gains increased with emission intensity and time, reaching 29.4% of the current area under SSP5-8.5 by 2081–2100. The spatial pattern of gains was predominantly upslope and along the cooler margins of the present range, consistent with thermal expansion of suitable terrain for a cold-adapted, high-altitude species as the plateau warms (Figure 5).

### 3.5. Uncertainty and Extrapolation

**Figure 6:**
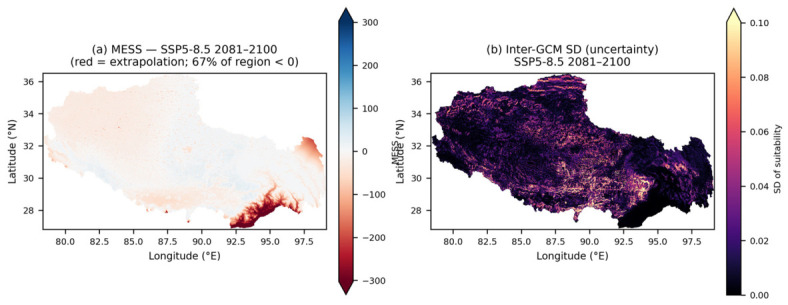
(**a**) MESS extrapolation surface for the worst-case scenario (SSP5-8.5 2081–2100, conservative union of two GCMs; 67% of region < 0); (**b**) inter-GCM standard deviation (uncertainty) surface for the same scenario.

**Figure 7:**
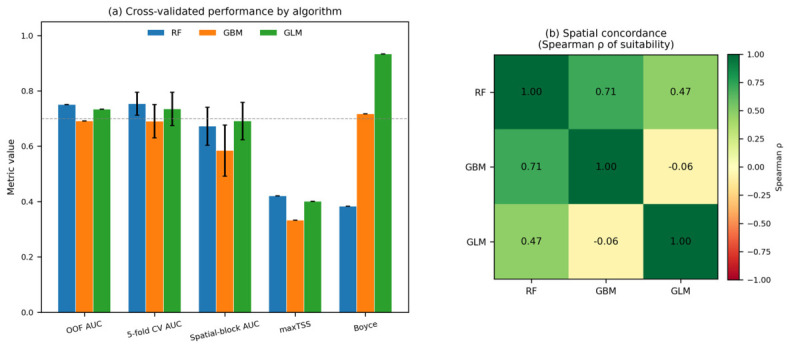
(**a**) Cross-validated performance (OOF, 5-fold, and spatial-block AUCs; maxTSS; Boyce) by algorithm; (**b**) spatial concordance (Spearman’s ρ of continuous suitability) between algorithm pairs.

Inter-GCM uncertainty was low, as the mean per-cell standard deviation of suitability within projected suitable areas ranged from 0.016 to 0.022 across all scenario–period combinations, and the two GCMs agreed on the suitable/unsuitable classification across the large majority of the suitable area (full-agreement areas in Table 4). However, MESS analysis revealed substantial climatic extrapolation, as 55–67% of the study region fell outside the environmental envelope defined by the 52 occurrence points in at least one predictor, increasing with emission intensity and time, and reaching 67% under the worst case (SSP5-8.5, 2081–2100; Figure 6). This is an expected consequence of modelling from a small, spatially restricted occurrence sample and projecting into novel plateau climates. It implies that, while the direction of change (net expansion via upslope gain) is robust and consistent across GCMs and scenarios, the precise magnitude of newly gained area is uncertain and should be interpreted as an upper-bound tendency rather than a calibrated area estimate. The per-algorithm suitability patterns and the cross-validated performance metrics that underpin these conclusions are shown in Figure 7.

## 4. Discussion

### 4.1. Upslope Expansion Contrasts with the Lowland Amphibian Paradigm

Most climate-change projections for amphibians forecast range contraction [7,23], consistent with the globally coherent fingerprint of climate change on natural systems [24], driven by warming beyond physiological optima, hydrological stress on breeding habitat, and the interaction of climate with disease and land-use change [25]. Our results for *N. parkeri* point in the opposite direction, as under every emission pathway and time slice, the bias-corrected ensemble projected a net expansion of climatically suitable habitat, reaching 117–126% of the current area by 2081–2100. The observed pattern of predominantly upslope gains mirrors elevational range shifts documented across diverse taxa [24,26]. This divergence is not paradoxical but is the expected signature of a cold-adapted, high-altitude species. For organisms currently constrained by low temperatures at the upper margin of habitable terrain, plateau warming releases that constraint and renders progressively higher, previously too-cold areas thermally suitable. The spatial pattern of gains—concentrated upslope and along the cool margins of the present range—is consistent with this mechanism, and the asymmetry of turnover (≈96% stable, only 3–5% lost, 17–29% gained) indicates that the present range is largely retained while new terrain is added at its cold edge.

This transient “climate-tracking upslope” benefit must be interpreted with three strong caveats. First, upslope expansion on a plateau is ultimately bounded by the finite and shrinking area of high terrain (the ‘elevational squeeze’ documented for mountain biota [26,27]); gains realised early in the century may give way to compression against the elevational ceiling under sustained warming, a dynamic our four-period horizon only begins to reveal (note the plateauing of expansion in the highest-emission scenarios late in the century). Second, and more fundamentally, climatic suitability is a necessary but not sufficient condition for habitat; *N. parkeri* is an obligate water-breeder, and our projections hold surface-water availability fixed at present-day values. Once the species’ climate envelope is pushed against the highest available elevations, the expansion phase may transition to one of contraction and population fragmentation, as suitable area is progressively compressed into smaller and increasingly isolated summit habitats—a dynamic our four-period horizon has not yet fully captured but that becomes more probable under sustained high-emission scenarios. Third, amphibian dispersal capacity is limited and breeding-site fidelity is often high [28]; the lag between the appearance of climatically suitable upslope terrain and actual colonisation may be measured in decades or longer, especially where topographic barriers or unsuitable matrix habitat separate current populations from newly suitable areas.

### 4.2. Hydrological Dependence May Decouple Climatic Suitability from Realised Habitat

The two hydrological-accessibility predictors (Section 2.3)—distance to lake and distance to river—together accounted for a Gini importance of 0.206, a substantial and independent contribution beyond the bioclimatic variables. This supports proximity to surface water as an important constraint on the distribution of *N. parkeri* over and above climate. The conservation implication is direct: a climatically suitable but waterless cell is not realised habitat. Our projections necessarily treat the hydrological layers as static because plateau-wide, scenario-specific future surface-water distributions are not reliably available at 30-arc-second resolution. Yet the Qinghai–Tibet Plateau is undergoing pronounced hydrological reorganization—glacier retreat, changes in lake area (with both expansion and contraction documented in different basins), permafrost degradation, and altered seasonal runoff [29,30]. If breeding waters at the newly thermally suitable upslope front fail to materialise, or if existing lowland waterbodies desiccate, the realised range expansion will fall short of the climatic projection. The gains we report should therefore be read as a climatic *envelope* of opportunity, conditional on hydrological persistence, rather than as guaranteed habitat.

### 4.3. Honest Metrics, Algorithm Robustness, and the Interpretation of Extrapolation

We deliberately report performance and projection diagnostics at face value rather than selecting the most favourable framing. The bias-corrected model’s out-of-fold AUC of 0.750 and spatially blocked AUC of 0.672 are modest, and considerably lower than the values an uncorrected, deeper model fitted with a random background would yield on the same data. We regard the lower numbers as the more honest estimate of the model’s transferable discriminatory ability; the target-group background removes the spurious “easy” contrast between sampled and unsampled geography, and spatial-block cross-validation removes the inflation that arises when spatially autocorrelated training and test points are intermixed.

Crucially, the broad, water-linked suitability surface is not an artefact of the random forest. Fitting a boosting ensemble (GBM) and a regularized linear model (GLM) on identical data and folds reproduced the broad suitable area (67–81% of the region), and the random forest tracked the three-algorithm ensemble closely (Spearman’s ρ = 0.742). Discrimination metrics varied more, as the GLM matched or exceeded the RF under spatial blocking (0.691 vs. 0.672), while the GBM had the lowest spatial-block AUC (0.584), a result that should be interpreted in light of its unweighted probability scale. The contrast in calibration is itself informative: the tree ensembles and the GLM emphasised different aspects of sparse presence–background structure, yet converged on the same regional conclusion. This strengthens confidence that the broad spatial pattern reflects the species–environment relationship rather than the inductive bias of one method.

The MESS analysis is reported in the same spirit. The finding that 55–67% of the projection region requires extrapolation beyond the environmental envelope of the 52 occurrence points is an unavoidable consequence of modelling a sparsely sampled species and projecting into novel plateau climates, and it would be misleading to present area forecasts without it. Critically, extrapolation and inter-model disagreement are distinct sources of uncertainty; the two GCMs agreed closely (per-cell suitability standard deviation 0.016–0.022, with broad full-agreement areas), so the *direction* of change—net upslope expansion—is robust to GCM choice. What extrapolation undermines is the *precision* of the gained-area magnitude, since the model is predicting into climate space it never saw during training. The defensible reading is qualitative confidence in the sign of the change and the spatial pattern of gains, coupled with explicit caution about the exact area numbers. Transparent reporting of extrapolation diagnostics alongside performance metrics, as recommended by recent SDM reporting standards [19], allows users to gauge projection credibility independently of summary accuracy statistics.

### 4.4. Limitations

Several limitations bound our conclusions. (1) The occurrence sample is small (n = 52 after thinning); although our methodological choices were designed to remain defensible at this size, the niche is necessarily characterized from limited environmental coverage, which is also the proximate cause of the high MESS extrapolation. (2) This is a regional assessment scoped to Xizang, chosen to align with the management jurisdiction; the species’ range extends beyond the region, so absolute suitable-area totals describe the Xizang assessment window rather than the entire range, and should thus be read as region-specific rather than range-wide figures. We accordingly emphasise the direction and spatial pattern of change, which are the quantities relevant to regional management. (3) Topographical and hydrological predictors were held static in projections; future changes in surface water and, to a lesser extent, in fine-scale habitat could alter the realised outcome, as discussed above. (4) The ensemble comprises two GCMs (BCC-CSM2-MR and MIROC6); while their agreement is reassuring, a larger ensemble would better sample structural climate-model uncertainty. (5) Projections assume full colonization of newly suitable cells (the “gain” class), i.e., dispersal is not explicitly modelled; for a low-vagility amphibian, realised colonization of the upslope front may lag the climatic opportunity, making our gain estimates an upper bound on near-term range change [28]. (6) Biotic interactions, disease (e.g., chytridiomycosis, which has caused catastrophic global amphibian declines [31,32,33] but whose distribution and impact at high elevations on the Qinghai–Tibet Plateau remain poorly characterised), and land-use changes are not incorporated. These limitations argue for treating the projections as scenario-conditional indicators of climatic opportunity rather than deterministic forecasts. (7) Long-term mean climatic variables—the standard in SDM practice—may fail to capture the effects of extreme events such as heat waves, prolonged droughts and anomalous precipitation pulses, to which amphibians can be acutely sensitive even when mean conditions remain within tolerance limits [25]. (8) High-elevation habitat suitability is modulated by factors beyond temperature and precipitation, including ultraviolet-B radiation intensity, snow-cover duration, dissolved oxygen in breeding waters, and prey (invertebrate) availability, none of which are explicitly represented in the predictor set. These unmodelled elevational covariates may impose additional upper limits on *N. parkeri*’s realised distribution. (9) Future work could narrow the gap between modelled climatic suitability and realised occupancy by incorporating fine-resolution habitat layers (e.g., wetland inventories from satellite imagery, land-cover classifications distinguishing breeding microhabitats), seasonal hydrological dynamics, species-specific physiological tolerance curves, and spatially explicit dispersal or metapopulation models that parameterise connectivity and colonisation lags.

### 4.5. Conservation Implications

Two spatial priorities emerge. First, the large stable core in Southern and Southeastern Xizang—river valleys and lake basins that remain suitable across all scenarios—constitutes the species’ climatic stronghold and warrants protection of its breeding waters as a no-regret conservation action. Second, the upslope colonization front, where climatic gains are projected, should be a focus of monitoring: confirming whether *N. parkeri* actually tracks suitability upslope, and whether breeding waters are present there, would directly test the central assumption of our projections. Because the species’ fate hinges on the persistence of surface water that our climate-only model cannot forecast, we recommend that climate-based suitability assessments for plateau amphibians be paired with explicit hydrological monitoring of glacier-fed streams, lakes and wetlands. More broadly, *N. parkeri* illustrates that “climate winners” among high-altitude ectotherms may be winners only in the narrow dimension of temperature, and remain vulnerable through the hydrological and elevational constraints that envelope models omit.

## 5. Conclusions

A bias-corrected species distribution model, robust across three contrasting algorithms, indicates that climatically suitable habitat for *Nanorana parkeri* is currently extensive (8.13 × 10^5^ km^2^) across Southern and Southeastern Xizang and is projected to expand upslope under all emission scenarios, reaching 117–126% of the current area by 2081–2100. This projected net expansion, driven by the warming-mediated release of cold constraints at high elevations, contrasts with the range contractions forecast for most amphibian taxa and positions *N. parkeri* as a potential transient “climate beneficiary” in the near term. However, three factors temper this optimistic projection: substantial climatic extrapolation (MESS 55–67%) limits the precision of area estimates; the finite area of high-altitude terrain ultimately bounds upslope expansion; and, most critically, the species’ obligate dependence on surface water means climatic suitability is necessary but not sufficient for realised habitat, while the plateau’s hydrology is itself being reorganized by glacier retreat, lake-area change, and altered runoff. Conservation priorities should focus on protecting the stable core breeding waters in Southern and Southeastern Xizang and on monitoring whether newly suitable high-elevation terrain is actually colonized and hydrologically viable. More broadly, this study underscores that climate-based assessments of high-altitude ectotherms must be paired with explicit hydrological monitoring, since “climate winners” in the temperature dimension may remain vulnerable through water and elevational constraints that envelope models omit. It is important to emphasise that these projections represent potential climatic suitability rather than predicted occupancy. The gap between climatic opportunity and realised distribution is mediated by dispersal limitation, breeding-site fidelity, fine-scale habitat filters, biotic interactions, and extreme climatic events—factors that our envelope model does not incorporate. The projected upslope expansion should therefore be interpreted as a scenario-conditional climatic envelope whose realisation depends on ecological and hydrological conditions beyond temperature alone.

## Figures and Tables

**Table 1:** Variable importance in the bias-corrected random forest model (nine predictors retained after VIF screening), ranked by Gini importance.

Variable	Description	Gini Importance
Bio12	Annual precipitation	0.145
Bio01	Annual mean temperature	0.131
Bio02	Mean diurnal range	0.124
Bio03	Isothermality	0.115
Dist_lake_km	Distance to lake	0.113
Bio15	Precipitation seasonality	0.103
Slope	Slope	0.096
Dist_river_km	Distance to river	0.093
Bio14	Precipitation of driest month	0.079

**Table 2:** Cross-validated performance and suitable area of three SDM algorithms fitted on identical data and folds. AUC values are out-of-fold (OOF) and 5-fold; spatial-block AUC uses 1° geographic blocks. Suitable area uses each algorithm’s own OOF-T10 threshold.

Algorithm	OOF AUC	5-Fold CV AUC	Spatial-Block AUC	maxTSS	Boyce	OOF-T10 Thr.	Suitable Area (km^2^)	% Region
Random forest	0.750	0.754 ± 0.041	0.672 ± 0.069	0.421	0.383	0.0276	813,027	67.5
Gradient boosting	0.692	0.690 ± 0.060	0.584 ± 0.093	0.333	0.717	0.0141	814,140	67.6
Regularized GLM	0.734	0.735 ± 0.060	0.691 ± 0.068	0.400	0.934	0.2316	978,610	81.2

**Table 3:** Spatial concordance between algorithm pairs: Spearman’s ρ of continuous suitability over all valid cells, and Jaccard index of binary suitable areas (each at its own OOF-T10 threshold).

Pair	Spearman’s ρ	Binary Jaccard
RF–GBM	0.708	0.648
RF–GLM	0.466	0.717
GBM–GLM	−0.063	0.595

**Table 4:** Ensemble (BCC-CSM2-MR + MIROC6 median) suitable area for *N. parkeri* by scenario and period, using the OOF-T10 threshold. Current suitable area = 8.13 × 10^5^ km^2^.

Scenario	Period	Suitable Area (km^2^)	% of Current	Full-Agreement Area (km^2^)
SSP1-2.6	2021–2040	916,267	112.7	811,235
SSP1-2.6	2041–2060	948,372	116.7	845,278
SSP1-2.6	2061–2080	956,551	117.7	858,825
SSP1-2.6	2081–2100	953,554	117.3	849,004
SSP2-4.5	2021–2040	929,222	114.3	815,018
SSP2-4.5	2041–2060	970,288	119.3	863,568
SSP2-4.5	2061–2080	993,857	122.2	897,149
SSP2-4.5	2081–2100	1,002,681	123.3	912,490
SSP3-7.0	2021–2040	916,124	112.7	796,906
SSP3-7.0	2041–2060	973,243	119.7	860,044
SSP3-7.0	2061–2080	1,010,238	124.3	917,049
SSP3-7.0	2081–2100	1,026,532	126.3	954,068
SSP5-8.5	2021–2040	925,877	113.9	815,454
SSP5-8.5	2041–2060	998,041	122.8	900,575
SSP5-8.5	2061–2080	1,027,749	126.4	956,268
SSP5-8.5	2081–2100	1,024,245	126.0	972,755

**Table 5:** Range turnover for representative end-of-century projections (2081–2100), as a percentage of current suitable area.

Scenario	Stable (%)	Lost (%)	Gained (%)
SSP1-2.6	96.4	3.6	20.9
SSP2-4.5	96.7	3.3	26.6
SSP3-7.0	97.2	2.8	29.1
SSP5-8.5	96.6	3.4	29.4

## Data Availability

The occurrence data, target-group background points, environmental predictor stacks, fitted model object, and replication Python code (version 3.13.12, Python Software Foundation, Wilmington, DE, USA) presented in this study are openly available in the Zenodo repository at https://doi.org/10.5281/zenodo.20806299 (accessed on 24 June 2026) under a Creative Commons Attribution 4.0 International (CC BY 4.0) licence. Third-party environmental datasets (WorldClim, HydroSHEDS) are available from their original providers under their respective licences.

## Supplementary Materials

The following supporting information can be downloaded at: https://www.mdpi.com/article/10.3390/ani16142169/s1, Table S1: Occurrence records of *Nanorana parkeri* used in species distribution modelling (52 records with geographic coordinates, elevation, data source, locality, and year).
